# Exploring dental professionals’ outlook on the future of dental care amidst the integration of artificial intelligence in dentistry: a pilot study in Pakistan

**DOI:** 10.1186/s12903-024-04305-7

**Published:** 2024-05-08

**Authors:** Wajiha Qamar, Nadia Khaleeq, Anita Nisar, Sahibzadi Fatima Tariq, Mehreen Lajber

**Affiliations:** 1 Department of Oral Biology at Bacha Khan College of Dentistry, Mardan, Pakistan; 2 Department of Community Dentistry, Institute of Public Health & Social Sciences, Khyber Medical University, Peshawar, Pakistan; 3 Senior Registrar at Department of Periodontology Rehman College of Dentistry, Peshawar, Pakistan; 4 Assistant Professor at Department of Oral Pathology Rehman College of Dentistry, Peshawar, Pakistan; 5 Department of Medical Education at Bacha Khan Medical College, Mardan, Pakistan

**Keywords:** Artificial Intelligence, Dentistry, Faculty, Pakistan, Education

## Abstract

**Objective:**

The purpose of this study is to explore the perspectives, familiarity, and readiness of dental faculty members regarding the integration and application of artificial intelligence (AI) in dentistry, with a focus on the possible effects on dental education and clinical practice.

**Methodology:**

In a mix-method cross-sectional quantitative and quantitative study conducted between June 1st and August 30th, 2023, the perspectives of faculty members from a public sector dental college in Pakistan regarding the function of AI were explored. This study used qualitative as well as quantitative techniques to analyse faculty’s viewpoints on the subject. The sample size was comprised of twenty-three faculty members. The quantitative data was analysed using descriptive statistics, while the qualitative data was analysed using theme analysis.

**Results:**

Position-specific differences in faculty familiarity underscore the value of individualized instruction. Surprisingly few had ever come across AI concepts in their professional lives. Nevertheless, many acknowledged that AI had the potential to improve patient outcomes. The majority thought AI would improve dentistry education. Participants suggested a few dental specialties where AI could be useful.

**Conclusion:**

The study emphasizes the significance of addressing in dental professionals’ knowledge gaps about AI. The promise of AI in dentistry calls for specialized training and teamwork between academic institutions and AI developers. Graduates of dentistry programs who use AI are better prepared to navigate shifting environments. The study highlights the positive effects of AI and the value of faculty involvement in maximizing its potential for better dental education and practice.

**Supplementary Information:**

The online version contains supplementary material available at 10.1186/s12903-024-04305-7.

## Introduction

The concept of artificial intelligence (AI) emerged as a breakthrough across various domains in an era of rapid technological advancement [1]. It has revolutionized industries and sectors around the world, aiming to create intelligent machines that can follow human cognitive capabilities. Its impact is remarkable, especially in the context of evolving global scenario. AI possesses the ability to improve diagnosis precision, effectiveness of treatment, and overall patient care, making healthcare one of the sectors where its significance is becoming more and more evident [2]. 

The application of AI into healthcare systems has already started transforming medical practices around the world through accurate diagnosis and tailored treatment plans [2]. In this regard, the prospect of AI cannot be overlooked in the field of dentistry, which is frequently viewed as an essential aspect of healthcare. Dental practitioners are currently employing AI-driven solutions to streamline the processes involved in diagnosis, treatment, and patient care [3]. AI assists with image analysis, predictive modelling, and decision support through various applications, improving clinical results [3]. 

Given its merits and use, the potential of AI in dentistry is particularly interesting. AI has proven to be helpful for addressing disparities in healthcare across a wide range of geographic and economic situations [4]. Delivering high-quality dental treatment to populations in countries that are developing like Pakistan presents particular difficulties. Due to its capacity to fill knowledge and scarcity of resources, AI now plays a more significant part in diagnostic and treatment planning [4]. AI can help dentists making informed choices that have a positive impact on patient outcomes by providing specific information and recommendations [5]. 

Additionally, it is impractical to overlook how AI is affecting both clinical practice as well as dental education. The incorporation of AI into the curriculum could provide fresh graduates the understanding and abilities they need to successfully navigate the changing dentistry practice landscape [6]. As a result, it is critical for dental faculty to comprehend, accept, and successfully incorporate AI concepts into their curricula. The inclusion of AI-related themes in the curriculum could enhance students’ ability to take leverage of technological advancements in their future careers, which will result in a more knowledgeable and skilled dental workforce [6, 7]. 

Dental faculty’s comprehension of AI is essential as the field of dentistry advances. However, it has been recognized that incorporating AI into dental education necessitates an appropriate approach [5]. In addition to having a thorough understanding of AI, faculty members must be able to develop and roll out programs that reflect how AI is employed in dental practice. Dental education demands a strategic capacity-enhancement program that can assist professionals negotiate the complex world of AI integration [6]. The faculty members who implement this program should be better able to understand the ethical implications, clinical significance, and practical application of AI in the field of dentistry.

Many studies are focusing on AI-powered solutions for automated detection of oral disorders, treatment planning optimization, and enhancement of interaction with patients as they explore the impact of AI on dentistry and dental education [3]. Furthermore, cooperative initiatives are being made to create thorough AI-focused curricula for dentistry schools, ensuring that future dental professionals have the knowledge to use full advantage of AI’s potential in their current positions. These works together add to the body of knowledge that emphasizes the revolutionary effects of AI in dentistry and emphasizes the importance of faculty involvement and comprehension of this development. Considering the existing knowledge and factors listed above, this study aims to review the dental faculty members’ perceptions and comprehension of the use of AI in dentistry. This study intends to provide insight into the current level of AI integration in dental education through studying the knowledge, attitudes, and readiness of faculty members to use AI. Additionally, it aims to emphasize the requirement for an established program for capacity training that enables dental faculty to successfully harness AI for the improvement of dental teaching and practice.

## Materials and methods

The cross-sectional quantitative study was carried out from 1st June to 30th August 2023 at a public sector dental college located in the Khyber Pakhtunkhwa province of Pakistan, which is recognized for graduating more than 175 students per year. Prior to commencing the study, approval was sought and obtained from the ethical review committee of the institute. The committee’s approval was obtained after presenting the objectives, methodology, and scope of the study for their review and consideration.

To ensure a representative sample, a simple random sampling technique was used. A comprehensive list of all prospective faculty members was compiled, encompassing individuals from various positions, including postgraduate trainees, lecturers, assistants, associates, and professors across both basic and clinical sciences departments of the dental college. Each prospective participant was assigned a unique number to facilitate randomization. Using a random number generator technique, 35 people at random were selected from the compiled list. Out of these 35 participants, only 23 participated in the study, yielding a response rate of 65.71%.

The study’s participation was voluntary, and the researchers individually interviewed those selected to ensure uniformity and consistency. Despite careful application of randomization to ensure an unbiased selection process, it is crucial to recognize the potential of selection bias resulting from voluntary participation. Self-selection bias may be introduced because individuals who freely choose to engage can have different perspectives or motives than those who decline. To minimize that, the researchers thoroughly explained the objectives and approach of the study during these interviews. Furthermore, participants’ rights and the study’s objective were properly disclosed, and ethical principles including informed consent were rigorously followed. Participants received assurance of anonymity, and individual privacy was protected through the aggregation and group presentation of data. In addition, participants were made aware that the data collected will be shared and used to guide policy choices, so advancing dental knowledge.

For this study, a specially designed questionnaire addressing a variety of topics about AI’s importance in dentistry was developed. The process of developing the questionnaire was informed by a thorough review of relevant research papers and articles in the domain. Rigorous efforts were made to validate the questionnaire’s relevance and appropriateness for the setting of research, reducing any concerns regarding its reliability, even though formal validation procedures were not feasible due to financial constraints. The purpose of the questionnaire was to gather opinions, knowledge, and suggestions from dental professionals about the use of AI in dentistry. Qualitative methods were also employed to augment the quantitative data that was gathered. Thematic analysis was conducted to analyse qualitative responses and further understand participant viewpoints.

Utilising statistical techniques, the quantitative data was gathered and analysed, enabling an in-depth analysis of participants’ perspectives about artificial intelligence in dentistry. The integration of quantitative and qualitative methodologies enabled a comprehensive investigation of faculty attitudes towards artificial intelligence in dentistry, resulting in a more comprehensive comprehension of the topic.

## Results

**Fig. 1:**
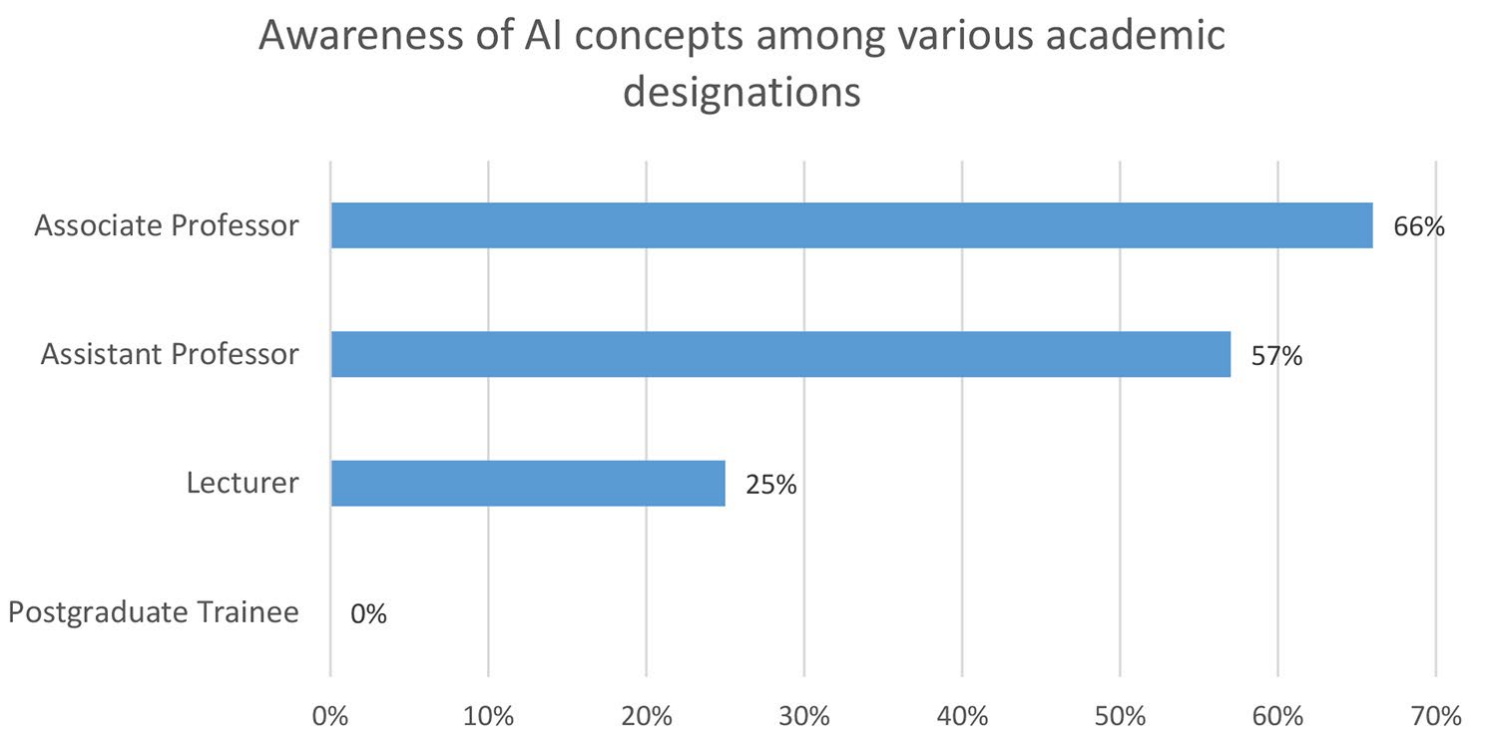
Awareness of AI concepts among various academic designations

Based on the findings from our study, we discovered that participants’ familiarity with AI ideas and their use in dentistry differed depending on their position. The overall awareness rate was 39.14% (*n* = 9), however there were notable variances when looked down by position. As shown in Fig. 1, while lecturers showed a familiarity rate of 25% (*n* = 3), postgraduate trainees claimed no familiarity. Among the participants, Associate Professors had the highest familiarity rate, at 66% (*n* = 3), while Assistant Professors displayed a slightly lower familiarity rate of 57% (*n* = 7). Further discussion and assessment revealed that only a small percentage of participants (8.7%, *n* = 2) reported having had any exposure to the concepts of artificial intelligence during their academic and professional careers. Participants with exposure to or understanding of AI concepts conveyed their perspective on AI application in dentistry, suggesting that integrating Computer-aided design (CAD) and Computer-aided manufacturing (CAM) and Exocad with AI-enabled Cone beam computed tomography (CBCT) interpretation as a promising way to improve the efficiency of diagnosis and treatment planning. They acknowledged these possible advantages, but they also recognised a lack of familiarity AI in dentistry and uncertain of its applicability or usefulness in their field. Nonetheless, the participants expressed a positive outlook about artificial intelligence’s potential to advance several facets of dental care, highlighting the technology’s ability to improve treatment planning and diagnostic accuracy, particularly about dentofacial abnormalities, orthodontic interventions, and prosthetic treatments.

On the other hand, none of the participants learned any AI principles while working in their respective fields. When explored they provided diverse perspectives about potential applications of AI in dentistry, highlighting the technology’s ability to facilitate treatment and planning procedures, patient record management, training medical and dental students, and microscope-assisted histology and pathology diagnosis. While some participants were uncertain or uninformed about AI in dentistry, others recognised how it may improve radiological results and treatment planning. They emphasized how it may help with a range of dental procedures, including orthognathic surgery, and increase the accuracy of diagnostics. It’s interesting to note that a sizable percentage of participants (82.6%, *n* = 19) recognized the value of AI applications in improving patient outcomes. The potential benefits of AI technology in dentistry were also emphasised by the participants such as to streamline diagnostic procedures, establish orthodontic and surgical treatment, and analyse radiological data. They also underlined the potential applications of AI in prosthetics, education, postoperative care, and preventative interventions. Some, meanwhile, voiced concerns or doubts about AI’s complete integration and efficacy in specific dental treatments.

Regarding the range of AI applications in dentistry, participants provided a range of viewpoints, from a targeted use in particular departments to a more general potential across the board. Some highlighted that AI is mostly useful in surgical settings, particularly for intricate extraction techniques. Others emphasised its importance in the planning of orthodontic treatments, where AI might improve accuracy and expedite procedures. Moreover, a portion of participants supported the use of AI in research projects, arguing that its analytical powers may help with data interpretation and analysis. On the other hand, other participants expressed their belief that AI technologies had the potential to completely transform the dental field. They envisioned AI being integrated into different practice areas and specialisations to maximise patient results and care. These different points of view highlight the continuous discussion in the dental community over the extent of AI’s influence and its potential to influence dentistry’s future.

Only 34.7% (*n* = 8) of the respondents said that basic sciences were another area where AI’s effectiveness was recognised, in addition to clinical sciences. During further discussion, it was revealed that the participants perceived that AI-driven educational technologies may be integrated into lectures to improve teaching methods, automated laboratory procedures can be used to accelerate studies, and AI can be used for microscopic examination and diagnosis of slides. Participants also emphasized how AI could enhance teaching and learning in dentistry education, especially in areas like anatomy and physiology, by using creative teaching strategies and interactive platforms.

**Fig. 2:**
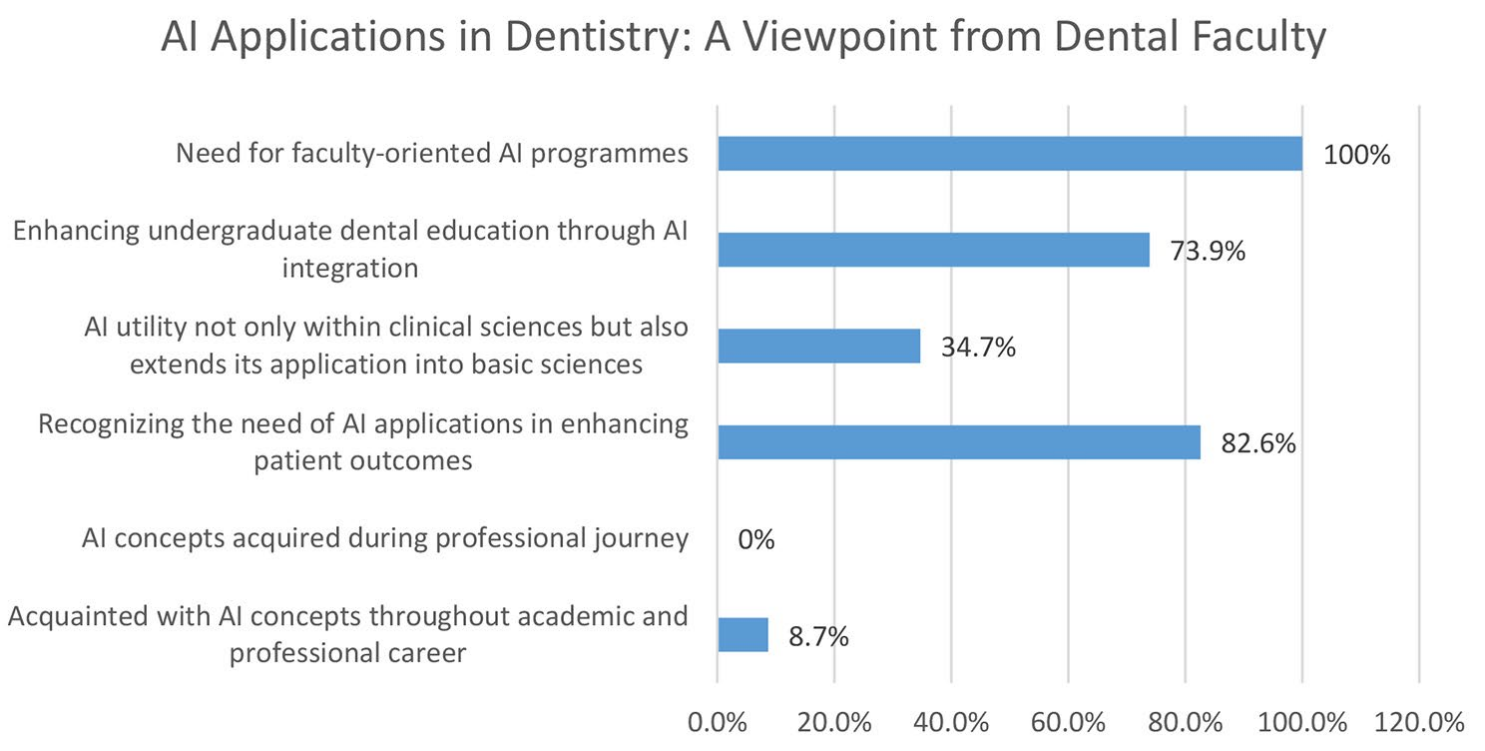
AI applications in dentistry: a viewpoint from dental faculty

Furthermore, a sizable majority (73.9%, *n* = 17) thought that the incorporation of AI may improve undergraduate dental education. The need for faculty-focused AI programs was acknowledged by everyone who participated. This highlighted the need for faculty in the field of dentistry to receive training that is specifically geared toward AI. A variety of topics and areas that participants felt should be included in a successful training programme were highlighted. These include the use of AI for research and undergraduate teaching, with an emphasis on the basic and clinical sciences. The significance of incorporating AI principles into methods of teaching, lectures, research projects, lab work, and clinical practice was also emphasised by the participants. A few responders also emphasised how important it is to comprehend the fundamental ideas behind AI and how it can be applied to certain fields like radiography, operational dentistry, and oral medicine. Fig. 2 provides an overview of AI applications in dentistry: A perspective of dental faculty.

**Fig. 3:**
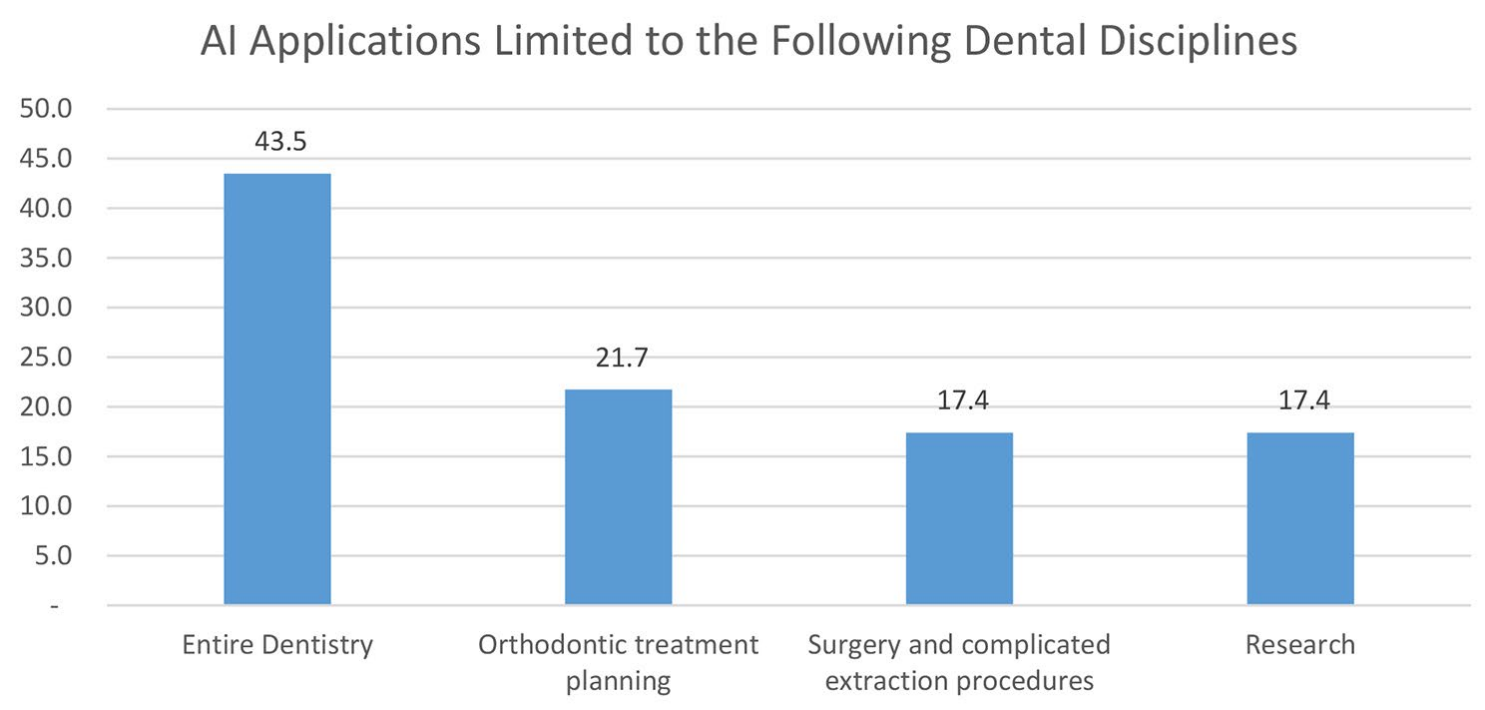
AI applications limited to the following dental disciplines

To ensure the efficacy of the tailored training programme in improving faculty competency in AI applications for dentistry, participants made several recommendations. One suggestion was to develop an extensive curriculum for undergraduate students that included AI’s function in both basic and clinical sciences. Additionally, they promoted the inclusion of AI principles in laboratory work, research, and education, with a focus on real-world applications. Participants also suggested specialised training courses centred on AI-related research, introducing pertinent software gradually after covering basic topics. In addition, suggestions included integrating AI training into faculty capacity development initiatives and holding interactive workshops and debates on the fundamentals of AI. The need of working with AI teachers was emphasised to gain more knowledge and understanding of how AI is used in dentistry. The perspectives of the participants areas of dentistry where AI are considered advantageous differed and are demonstrated in Fig. 3. One of the many fields where AI has the potential to be useful in the future is “entire dentistry”, according to 43.5% (*n* = 10) of participants. However, 21.7% (*n* = 5) of respondents specifically mentioned that the use of AI is limited to orthodontic treatment planning, while 17.4% (*n* = 4) of respondents added that AI might only be useful for difficult surgical and extraction procedures.

These results highlight the diverse levels of understanding of artificial intelligence (AI) concepts among dental specialties, the recognition of AI’s potential to enhance patient outcomes, and the significance of incorporating AI into dental education and clinical practice. The study also identifies several dental fields where AI is thought to have significant impact.

The findings of the chi-square test suggest a significant association between participants’ positions and their familiarity with AI concepts (χ² = 37.21, df = 3, *p* < 0.001). This underscores the importance of considering professional roles when exploring attitudes and understanding towards AI in dentistry.

## Discussion

We investigated dental faculty knowledge, exposure to, and attitudes regarding AI in the field of dentistry. The findings provided interesting insights concerning the interactions of these professionals in different roles with AI concepts.

The results showed significant variations in positions’ familiarity with AI. The associate professors displayed the highest familiarity, at 66%, while lecturers had the lowest, at 25%, emphasizing the importance of experience. The lack of familiarity indicated by postgraduate trainees highlights educational gaps. Our results are consistent with similar research from an adjacent region, which identified similar patterns of low AI comprehension among dental professionals [8–10]. To address the awareness disparity, both studies underlined the necessity for AI education and its integration with dental courses [9]. 

Additionally, just 8.7% of our indicated they had encountered AI concepts at some point in their academic or professional careers. Despite several confessing to being inexperienced with the AI, participants cautiously see potential in AI for dentistry, notably in enhancing diagnostic and treatment planning [10]. Our study emphasizes the significance of comprehensive AI education integration into dental courses and ongoing professional development programs [10]. Furthermore, given that AI concepts are seldom taught in the workplace, it raises concerns about how effectively society will be able to adopt new technology. Most communication channels will eventually be transformed by AI, necessitating the need for dental schools to educate current students about these significant advances in technology [11]. In addition to opening opportunities to train dental professionals to employ AI approaches in clinical practice, its adoption holds huge potential for improving healthcare and educational opportunities [6, 12]. The dental community and regulatory authorities must implement the suggested criteria to properly digitalize dentistry education with AI technology. Research projects that could involve academic, business, and government collaboration can improve knowledge and use of AI education in the workplace.

Considering the rapid advancement of AI in healthcare sectors, including dentistry, this lack of exposure is particularly alarming. For procedures like diagnostics, individualized treatment plans, and predictive analytics, other sectors have embraced AI [3]. The incorporation of AI in the field of dentistry in blooming in recent years, unlike other industries [13]. 

Despite having minimal exposure to and knowledge with AI, a significant percentage of participants (82.6%) were aware of its potential to enhance patient outcomes. This implies a theoretical comprehension of the advantages of AI, even though it might not directly translate to implementation in practice. This confidence in AI’s potential to improve patient outcomes is consistent with findings from related studies in the medical field [4, 6, 13, 14]. A recent study by Bajwa et al. (2021) found that a sizable percentage of healthcare professionals believe AI could contribute to better patient care. However, our study adds depth by exploring the differences in familiarity based on various positions within the dental profession.

Surprisingly, our study revealed a positive assessment of AI’s contribution to dental education, despite participants’ limited understanding and background in AI concepts. Most participants (73.9%) thought that using AI may improve undergraduate dental education. This finding supports the growing body of evidence that shows AI has the capability to transform education through targeted instruction, data-driven insights, and interactive simulations [4, 9, 13, 15]. 

Only 34.7% of the participants thought that AI could be utilized in basic sciences, demonstrating the need for a greater awareness of AI’s more general applicability. These concerns are similar to those raised in research that revealed challenges in describing the transdisciplinary character of AI [9]. To fully embrace AI’s potential, professionals must understand how it may be applied across a variety of fields.

The potential of AI in dental education has been highlighted in studies, which is consistent with our participants’ positive thoughts toward its incorporation into undergraduate the dental profession education [16]. However, our work adds to this body of knowledge by highlighting the necessity of faculty members receiving specialized AI training to ensure successful implementation.

One of the most important conclusions from our study is that all participants acknowledged that faculty-focused AI training programs are essential. This emphasizes how crucial it is for academics to be knowledgeable about AI to integrate it into the curriculum and educate a new generation of AI-savvy the dental professionals. Tailored training programmes that meet the unique demands of different academic levels and are based on in-depth assessments of training needs are crucial. These courses ought to be designed to improve their capacity to use AI to dentistry in a productive way.

Our study distinguishes itself by focusing on the position-based variations in AI familiarity when comparing our results with those of earlier studies carried out in comparable regions and among comparable populations. Our research stands out from others since we have concentrated on the critical requirement for faculty development that is specifically geared toward AI.

While highlighting current circumstances, our study additionally highlights the significance of ongoing research to monitor the progression of AI awareness and integration in dentistry. An ongoing assessment of dental professionals’ readiness to use AI technologies is critical as these technologies advance. Following the implementation of faculty-focused training programs, this can entail conducting follow-up research to evaluate changes in familiarity rates, perceptions, and real AI integration.

## Conclusion

The study’s conclusion underlines that there exists significant difference in familiarity with AI concepts among dental professionals based on their respective positions within the profession. Despite varying levels of familiarity, a consensus among participants acknowledges the potential of AI applications in improving patient outcomes. There may be a training and education gap in dentistry given the limited exposure to AI concepts during academic and professional careers. Therefore, it’s imperative to develop a comprehensive curricula that highlight real-world applications and integrate AI’s role in both basic and clinical sciences for undergraduate students.

The integration of AI is seen advantageous in several dental fields, such as clinical practice, fundamental sciences, and dental education for undergraduates. This calls for, applying AI concepts to research, teaching, and lab work while emphasising useful applications and real-world situations. Moreover, all the participants concur that faculty-focused AI programmes are essential, highlighting the need of specialised training programmes to equip dental professionals for the integration of AI into clinical practice and instruction such as interactive seminars and debates to foster understanding as well as AI training in faculty capacity building programmes. Lastly, it is crucial to provide specialised training classes on AI-related research; after going over the fundamentals, progressively introduce relevant software.

## Limitations

It’s critical to recognise several limitations when assessing the implications of our study’s findings since they might affect how the data are interpreted and used more broadly. Among these some are:

1. The limited sample size of the study, which originated from a single dental institution in a specific region of Pakistan, could make it challenging to generalise the results to a wider population of dental professionals.
2. Due to the voluntary nature of participation, selection bias may be introduced, which might affect participant perspectives and attitudes as well as the sample’s overall representativeness.
3. The study’s cross-sectional approach makes it difficult to establish causal links and makes it difficult to observe changes in views regarding artificial intelligence in dentistry over time.

### Electronic supplementary material

Below is the link to the electronic supplementary material.

Supplementary Material 1

## Data Availability

The datasets generated and/or analysed during the current study are not publicly available as we assured all participants that their data would be handled with the utmost confidentiality and under no circumstances would it be shared with any other parties. However, the datasets created and/or analysed during the current study are not publicly available but are nonetheless available from the corresponding author upon reasonable request.
