# Supplementary material for: Exploring dental professionals’ outlook on the future of dental care amidst the integration of artificial intelligence in dentistry: a pilot study in Pakistan
Source: BMC Oral Health. 2024 May 8;24:542. doi: 10.1186/s12903-024-04305-7 (PMC11080197; doi:10.1186/s12903-024-04305-7)
Supplement: Supplementary file 1 — Supplementary Material 1 [file 12903_2024_4305_MOESM1_ESM.docx]

**Questionnaire**

| Thank you for participating in this study, which aims to gain an insights into your perspectives and knowledge concerning the application of Artificial Intelligence (AI) in dentistry. By completing this self-administered questionnaire, you hereby provide your consent to participate.  The authors of this study are committed to maintaining the confidentiality of your identity. Your individual identity will remain undisclosed, and the information you provide will be aggregated for presentation in group data. This aggregated information will aid decision-makers in tailoring initiatives related to AI implementation.  Your valuable insights will play a crucial role in shaping the development of a customized training program. This program aims to enhance the capacity of faculty members in embracing AI advancements within the field of dentistry.  Kindly respond to the questionnaire based on your own experiences and perspectives. Your participation will greatly contribute to the advancement of knowledge in this domain. |
| --- |

1. Name (Optional): _____________________ 2. Cell Number: ________________________________
2. Designation (Encircle One): a). PG Trainee b). Lecturer c). Assis. Prof d). Assoc. Prof

e). Professor

1. Dental Speciality: ____________________________
2. Years of Experience in Dental Education (after house job): ____ Year
3. Are you familiar with the concept of "Artificial Intelligence" (AI) and its application in dentistry? [ ] Yes [ ] No
4. Please provide a brief description of your understanding of how Artificial Intelligence (AI) can be applied in the field of dentistry ____________________________________________________________________________________

______________________________________________________________________________________________

1. Have you encountered AI-related concepts or technologies in the field of dentistry during your educational or professional journey? [ ] Yes [ ] No
2. If you answered "Yes" to the previous question, please provide a brief description of any specific AI-related concepts or technologies you have come across in dentistry_____________________________________________________

______________________________________________________________________________________________

______________________________________________________________________________________________

1. Have you undertaken any courses on the concepts and applications of AI in the field of dentistry? [ ] Yes [ ] No
2. How do you see the potential of AI in dental diagnosis and treatment planning? Please provide examples if possible ______________________________________________________________________________________________

______________________________________________________________________________________________

______________________________________________________________________________________________

1. Do you believe that AI applications could contribute to improving patient outcomes in dental practice? [ ] Yes [ ] No
2. In your view, what aspects of dentistry could be positively impacted by the use of AI technologies? Please elaborate.

_______________________________________________________________________________________________

_______________________________________________________________________________________________

1. How would you rate your current understanding of AI applications in dentistry? [ ] Confident [ ] Not Confident
2. Do you perceive AI applications are limited to specific departments of dentistry or applicable across the entire field? Please share your understanding __________________________________________________________________

_____________________________________________________________________________________________

1. Can AI concepts and application be employed in the Dental Basic Sciences? [ ] Yes [ ] No
2. If you answered "Yes" to the previous question, please provide a brief description on how ____________________

______________________________________________________________________________________________

______________________________________________________________________________________________

1. Do you see the relevance of applying AI in the education of undergraduate dental students? [ ] Yes [ ] No
2. Have you ever attempted to incorporate AI-related concepts into your teaching or lectures? [ ] Yes [ ] No
3. If you answered "Yes" to the previous question, please briefly describe your experience in using AI concepts into your teaching ______________________________________________________________________________

______________________________________________________________________________________________

1. How receptive do you believe dental students would be to learning about AI concepts as part of their dental education? [ ] Receptive [ ] Not Receptive
2. Do you believe that a training program on AI applications in dentistry would be valuable for enhancing faculty capacity? [ ] Yes [ ] No
3. If Yes, what specific areas or topics do you think the training program should cover to be effective? _____________

_______________________________________________________________________________________________

_______________________________________________________________________________________________

1. Would you be interested in participating in a training program focused on enhancing your understanding of AI applications in dentistry? [ ] Yes [ ] No
2. Based on your understanding, what recommendations would you suggest to ensure that the customized training program effectively enhances faculty understanding and capacity in AI applications for dentistry? _______________

_______________________________________________________________________________________________

_______________________________________________________________________________________________

Thank you for your valuable participation in this study. Your responses will provide a comprehensive view of faculty understanding, behaviour, and capacity for transferring AI concepts in the field of dentistry. This information will greatly contribute to the development of a tailored training program that enhances faculty engagement with AI concepts and applications.
